# Using TRIS-Buffered Plasma-Activated Water to Reduce Pathogenic Microorganisms on Poultry Carcasses with Evaluation of Physicochemical and Sensory Parameters

**DOI:** 10.3390/foods12051113

**Published:** 2023-03-06

**Authors:** Vanessa Große-Peclum, Lisa Siekmann, Carsten Krischek, Georg Avramidis, Christian Ochs, Wolfgang Viöl, Madeleine Plötz

**Affiliations:** 1 Institute of Food Quality and Food Safety, University of Veterinary Medicine Hannover, Foundation, 30173 Hannover, Germany; 2 Faculty Engineering and Health, University of Applied Sciences and Arts, 37085 Göttingen, Germany

**Keywords:** plasma-activated water, cold plasma, TRIS-buffer, poultry skin and meat, *Escherichia coli*, *Campylobacter jejuni*, food safety, food quality

## Abstract

Foodborne diseases are mainly caused by the contamination of meat or meat products with pathogenic microorganisms. In this study, we first investigated the in vitro application of TRIS-buffered plasma-activated water (Tb-PAW) on *Campylobacter (C.) jejuni* and *Escherichia (E.) coli*, with a reduction of approx. 4.20 ± 0.68 and 5.12 ± 0.46 log_10_ CFU/mL. Furthermore, chicken and duck thighs (inoculated with *C. jejuni* or *E. coli*) and breasts (with natural microflora) with skin were sprayed with Tb-PAW. Samples were packed under a modified atmosphere and stored at 4 °C for 0, 7, and 14 days. The Tb-PAW could reduce *C. jejuni* on days 7 and 14 (chicken) and *E. coli* on day 14 (duck) significantly. In chicken, there were no significant differences in sensory, pH-value, color, and antioxidant activity, but %OxyMb levels decreased, whereas %MetMb and %DeoMb increased. In duck, we observed slight differences in pH-value, color, and myoglobin redox forms for the Tb-PAW, which were not perceived by the sensory test persons. With only slight differences in product quality, its application as a spray treatment may be a useful method to reduce *C. jejuni* and *E. coli* on chicken and duck carcasses.

## 1. Introduction

Worldwide, pathogenic microorganisms contaminate meat or meat products, posing a serious health risk to humans [1]. The poultry meat produced in the European Union in 2020 consisted, among others, of 82% broiler meat, 14% turkey meat, and 3% duck meat. Accordingly, the largest part of consumption is broiler meat [2]. However, in other countries, especially in the South Asian region such as Korea, the consumption of duck meat and duck meat products has increased sharply, about five times between 1997 and 2012 [3]. *Campylobacter jejuni* (*C. jejuni*) is considered to be the most common bacterial cause of humans gastroenteritis (campylobacteriosis) in the world [1]. In Germany, campylobacteriosis is mainly caused by ingestion of the pathogen via raw and insufficient heated meat, unpasteurized milk (raw milk), or, less frequently, through contaminated drinking water [4]. In isolated cases, *C. jejuni* can also cause life-threatening diseases such as Guillain-Barré syndrome, which involves neuro-muscular paralysis [5]. *C. jejuni*—which occurs in the intestinal tract of poultry—and other foodborne pathogens such as *Escherichia coli (E. coli)*, *Listeria monocytogenes*, or *Salmonella* spp. can be transferred to carcasses during the slaughtering process [6,7]. In chicken slaughtering, defeathering and evisceration are the most critical process steps for contaminating the meat with *C. jejuni* or *E. coli* [8].

Many scientists have already tried various decontamination strategies to reduce the bacterial load of carcasses and achieve a simultaneous improvement in food shelf life, such as using chemicals (ethyl-Nα-lauroyl-L-arginate hypochloride, peracetic acid, or chlorine-based products), UV-C radiation, or different temperature regimes [9,10,11,12]. Unfortunately, these applications often cause negative effects on the products, such as damage to texture, alteration of taste, or formation of carcinogens [13]. Only the use of potable water to remove surface contamination on poultry meat is currently permitted due to European Regulation (EC) No. 853/2004 (Article 3(2)) [14].

In recent years, the use of cold atmospheric plasma (CAP) to reduce a variety of microorganisms on meat and meat products has become increasingly important [15]. However, Rahman et al. [13] described that the design of the plasma devices (mainly atmospheric pressure plasma jet or dielectric barrier discharge) results in decontamination of only a part and not the whole food surface, especially for larger products. In addition, they found that the microorganisms are not affected by the plasma in some cases due to the shielding effect of the surface structure and texture of skin or meat.

Plasma-activated liquids (PAL) have the potential to decontaminate the surface of food [16,17] and contribute to the minimization of pathogens and prevention of human foodborne illnesses [18]. PALs can be made from a variety of liquids, including pure distilled water (plasma-activated water; PAW), phosphate-buffered saline, citrate-phosphate buffers, or acetic acid [19,20,21]. They contain various highly reactive oxygen and nitrogen species (RONS), such as hydrogen peroxide (H_2_O_2_), hydroxyl radicals (OH^–^), peroxynitrite (ONOO^–^), or nitrogen dioxide (NO_2_^–^), which are expected to be the key species responsible for the antimicrobial activity [22,23].

Some PALs have already been evaluated for their ability to decontaminate food (vegetables, fruits or meat), with promising results [16,24]. For example, Xiang et al. [24] showed that a 30 min PAW treatment of mung beans could reduce total aerobic bacteria by approx. 2.32 CFU/g. Ma et al. [16] were the first to treat strawberries with PAW and achieved a reduction of approx. 3.4 log_10_ CFU/mL. However, with regard to the application of PAW for decontamination on poultry skin or meat, only a few studies have been conducted so far [17,25]. Kang et al. [25] showed that PAW could effectively reduce *Pseudomonas deceptionensis* CM2 on chicken meat by approx. 1.05 log_10_ CFU/g, but was associated with significant impairment of chicken breast appearance, odor, texture, and acceptability compared with untreated samples. The reductive properties as well as the influences on meat quality by PALs may vary. This may be due to the fact that different production parameters such as voltage, working gases and gas flow rate, liquid used, or plasma treatment time are applied [17,26,27,28].

In this study, we investigated the effect of a TRIS-buffered PAW (Tb-PAW) on *C. jejuni*, *E. coli* and total viable count (TVC) of chicken and duck skin (natural matrix) immediately and after 7 and 14 days of storage, respectively. Following microbiological testing, physicochemical parameters such as antioxidant activity, myoglobin redox forms, and pH-values of meat and skin, as well as sensory parameters were analyzed. These studies will provide information whether Tb-PAW has the potential to reduce contamination on carcasses in meat processing. This should prevent the transmission of pathogens to humans. In addition, this study will investigate the effects of Tb-PAW on the shelf life of meat products and visible sensory characteristics of the meat and skin.

## 2. Materials and Methods

### 2.1. Production of Tb-PAW

**Figure 1:**
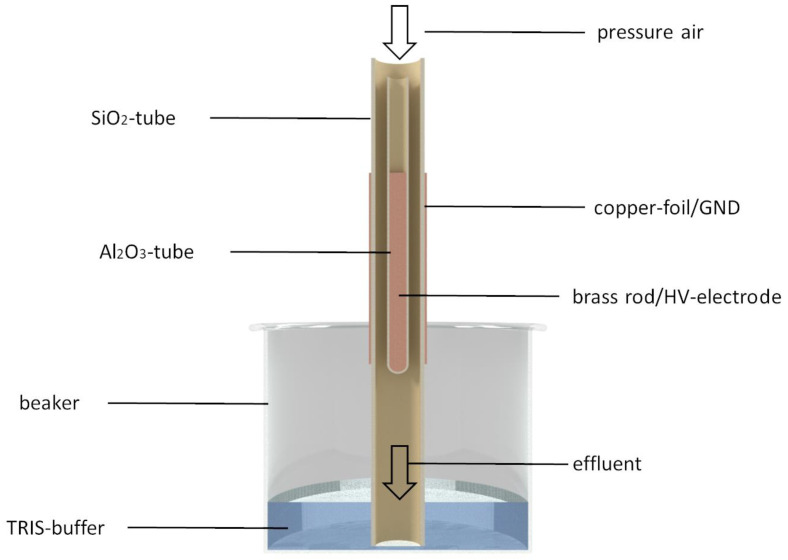
Scheme of a single plasma tube to generate Tb-PAW.

For this study 300 mL of a fresh TRIS-buffer (0.5 mol/L), made out of TRIS (TRIS(hydroxymethyl)aminomethan, Trometamol, ≥99.8%, VWR International, Darmstadt, Germany) and TRIS HCl (TRIS(hydroxymethyl)aminomethan hydrochlorid, ≥99.0%, VWR) were used. Tb-PAW was prepared using 20 min activation by plasma at the Faculty of Engineering and Health (HAWK, University of Applied Science and Arts, Göttingen, Germany) using a PAL-reactor based on the principle of a double-insulated dielectric barrier discharge. The setup for the generation of Tb-PAW consists of an array of 10 plasma tubes. The single plasma tube consists of an outer silica tube (l = 300 mm, outer diameter 12 mm, wall thickness 1 mm, inner diameter 10 mm), wrapped with copper-foil as ground electrode (GND) and a centrally positioned Al_2_O_3_-tube (outer diameter 3 mm, inner diameter 1.6 mm) filled with a brass rod acting as high-voltage electrode (HV-electrode). The discharge gap (length 100 mm) is streamed with pressure air at a gas flow rate of 5 L min^−1^. The outer quartz tube protrudes approx. 5 cm into a beaker filled with the TRIS-buffer (Figure 1).

The high voltage power supply provides alternating pulses (U = 16.6 kV peak-peak, f = 17 kHz, t_pulse_ = 2 µs) with an in-coupled power of approx. 400 W to the array. The device used for Tb-PAW production and the corresponding process parameters have already been published by Große-Peclum et al. [29], where a more detailed description of the configuration and operating parameters can be found (see also Supplementary Materials). The experiments started within 4 to 5 h after preparation and transportation to the Institute for Food Quality and Food Safety (LMQS, University of Veterinary Medicine Hannover Foundation, Germany). For the experiments, the pH-values of the TRIS-buffer and Tb-PAW were measured with a pH-meter (Jenway, Cole- Parmer, Stone, Staffordshire, ST15 OSA, UK). Due to the pH decrease of the Tb-PAW (7.3 ± 0.2), the pH of the TRIS-buffer (7.6 ± 0.2) was adjusted in order to exclude a pH effect.

Using a Reflectoquant (Merck KGaA, Darmstadt, Germany), the Tb-PAW was analyzed with regard to the concentrations of nitrate (NO_3_^−^; approx. 5540 mg/L), nitrite (NO_2_^−^; approx. 440 mg/L), as well as hydrogen peroxide (H_2_O_2_; approx. 4.5 mg/L) on each experimental day.

### 2.2. Bacterial Strains and Culture Conditions

*E. coli* (DSM 682) and *C. jejuni* (DSM 4688) were obtained from the German Collection of Microorganisms and Cell Cultures GmbH (DSMZ, Braunschweig, Germany). The microorganisms were plated on Columbia blood agar with sheep blood (Oxoid GmbH, Wesel, Germany). *E. coli* was incubated aerobically at 37 °C for 24 h, whereas *C. jejuni* was incubated at 41.5 °C under microaerophilic conditions (5% O_2_, 10% CO_2_, and 85% N_2_). A stock culture from each bacterial strain was maintained in a cryotube (Carl Roth, Karlsruhe, Germany) at −80 °C. For the experiment, bacterial colonies were suspended in sterile saline (0.9% NaCl). Subsequently, *E. coli* was adjusted to a McFarland turbidity standard of 1.5 and *C. jejuni* to 3.0 (approx. 10^7^–10^8^ CFU/mL).

### 2.3. Tb-PAW Treatment of E. coli and C. jejuni

The microbiological tests were performed in accordance with ISO-10272-1 and ISO-16649-2. 9 mL of Tb-PAW and 9 mL of TRIS-buffer (control) were added to sterile test tubes. Then 1 mL of each bacterial suspension (approx. 10^7^–10^8^ CFU/mL) was added to the Tb-PAW or TRIS-buffer, vortexed, and incubated for 1 min at room temperature. This was followed by serial dilutions, and 100 µL of the appropriate dilution stage was spread in duplicate to Colic Brilliance™ *E. coli*/coliform selective agar (ColiC agar, Oxoid, for *E. coli*) or CCDA selective agar (CCDA, Oxoid, for *C. jejuni*). Agar plates were incubated as already described (Section 2.2).

### 2.4. Sample Preparation

For the experiments, 18 fresh chicken carcasses each were obtained from a poultry slaughterhouse and 18 fresh duck carcasses each from a local market in three independent replicates. The carcasses were immediately transported to the LMQS under cooled conditions and stored at 4 °C for one day. The experimental procedure for the chicken and duck carcasses was comparable. Each carcass was dissected to obtain two breasts and two thighs with skin. The breasts and thighs were first weighed (chicken breasts 379 ± 24 g and chicken thighs 318 ± 21 g; duck breasts 376 ± 12 g and duck thighs 345 ± 90 g) and then placed in trays (polypropylene; ES Plastic GmbH & Co. KG, Passau, Germany). Half of the skin of each breast was removed to study the TVC and the presence of *E. coli* and *C. jejuni*, as well as to analyze the effects of Tb-PAW and TRIS-buffer on meat quality directly (skin was previously removed) and indirectly (meat under the skin). Subsequently, 18 thighs were inoculated with 100 µL (approx. 10^4^–10^5^ CFU/mL) of the *E. coli* suspension, whereas *C. jejuni* was distributed on the other 18 thighs. After inoculation, the samples were stored at 4 °C for 30 min to allow bacterial attachment before Tb-PAW treatment. Breast skins were not inoculated and were used to evaluate the effect of the Tb-PAW treatment on TVC.

### 2.5. Tb-PAW Treatment and Storage Conditions

For spraying, both Tb-PAW and TRIS-buffer were filled into a universal sprayer from retail. The surface of the breasts and thighs were sprayed with 3 mL ± 0.5 Tb-PAW or TRIS-buffer (control) from a distance of 15 cm, resulting in a surface density of approx. 11 µL/cm². Untreated breasts and thighs were used as additional controls. After spraying, the trays were filled with 70% N_2_ and 30% CO_2_ and sealed with a transparent film in a semi-automatic packaging machine (Multivac T100, Sepp Haggenmueller GmbH & Co. KG, Wolfertschwerden, Germany). After packaging, the samples were stored at 4 °C for 0, 7, and 14 days. The samples from day 0 were analyzed on the day of packaging, the others on days 7 and 14.

### 2.6. Sensory Evaluation

Three test persons using a 5-point scale evaluated the sensory characteristics of the appearance and odor of the products immediately after opening. The scores were 5 for very good—no deviation from quality expectations, 4 for good—minor deviations, 3 for satisfactory—moderate deviations, 2 for less satisfactory—significant deviations, and 1 for unsatisfactory—major deviations. For the result, following the DLG 5-point test scheme^®^ the points for appearance were multiplied by 3, added with the points for smell and the sum divided by 4 [30].

### 2.7. Microbiological Analysis

Microbiological examinations were performed according to ISO 4833-1:2013. A 5 g skin sample was collected from the breasts and thighs of each treatment group (Tb-PAW, TRIS-buffer, untreated), placed in a sterile bag, filled up to 50 g with peptone-buffered saline (0.85% NaCl, 0.1% peptone) (VWR), and then homogenized in a Stomacher R© 400 Circulator (Seward Ltd., Worthing, United Kingdom) for 2 min at 230 rpm. Serial dilutions were performed by adding 1 mL of the sample solution to 9 mL of peptone-buffered saline. Subsequently, 100 µL of the diluted suspension was plated on ColiC agar for detection of *E. coli* and on CCDA for detection of *C. jejuni*. For TVC, 1 mL of the suspension was pipetted into a petri dish and filled with warm plate count agar (Oxoid). After incubation (*E. coli* 24 h at 37 °C; *C. jejuni* 48 h at 41.5 °C; TVC 72 h at 30 °C), the number of colonies per plate were counted. The results are expressed as log_10_ CFU/g skin.

### 2.8. Color Measurement

After opening the packages containing the breast samples, the surface color of the skin, the skinless meat (directly, skin was previously removed, described in Section 2.4), as well as the meat under the skin (indirectly) were measured using a Chromameter (Minolta CR-400, Konica-Minolta GmbH, Langenhagen, Germany). Using the CIE L*a*b* system, five measurements per sample were taken, and the average of these repeats was used for further statistical analysis. L* stands for lightness/darkness, a* for redness/greenness, and b* for yellowness/blueness.

### 2.9. pH-Value Measurement

After color measurement, the pH-values of the chicken breasts (treatment groups: Tb-PAW, TRIS-buffer, untreated) were measured. A portable pH-meter equipped with a glass electrode (InLab 427 R©, Mettler-Toledo, Urdorf, Switzerland) and a thermometer (Knick Portamess, Knick GmbH, Berlin, Germany) was used for this purpose. Both the glass electrode and the thermometer were inserted into the muscle for measurement.

### 2.10. Analysis of Antioxidant Activity

For the analysis of antioxidant activity, a 1 cm layer was carefully removed from the breast muscle surface below the treated skin, cut into small pieces and stored at −80 °C until analysis (treatment groups: Tb-PAW, TRIS-buffer, untreated). According to Re et al. [31], a 2,2′-azinobis(3-ethylbenzothiazoline-6-sulfonic acid) diammonium salt (ABTS^·+^) radical solution was prepared by reacting a 7 mM ABTS solution (ABTS dissolved in distilled water) with potassium persulfate. The radical solution was incubated in the dark for 12 to 16 h at room temperature. Prior to analysis, the ABTS^·+^ radical cation was adjusted with distilled water to an absorbance of 0.70 ± 0.02 at 734 nm. Then 1 g of a breast meat sample was homogenized on ice with 6 mL of distilled water (1 min at 30.000 rpm) and shaken for 1 h at 4 °C in the dark. After centrifugation of the homogenate (2340× *g*; 15 min; 4 °C) 20 µL of the supernatant was added to 3 mL of ABTS^·+^ radical solution. The same amount of distilled water was used as a control. After 7 min of incubation, the absorbance was measured spectrophotometrically (Evolution 201-UV-VIS spectrophotometer, Thermo Scientific, Waltham, MA, USA) at 734 nm. For calculation of the antioxidant activity, a linear standard curve with 6-hydroxy-2,5,7,8-tetramethychroman-2-carboxylic acid (Trolox) and R^2^ > 0.99 was considered. For creation of the curve, 20 μL of 5 standard solutions containing 2.5, 5.0, 7.5, 10, and 15 μM Trolox were added to 3 mL of ABTS^·+^ radical solution and analyzed as described above.

### 2.11. Analysis of Myoglobin Redox form Percentages

For the analysis of the myoglobin redox forms deoxymyoglobin (DeoMb), oxymyoglobin (OxyMb), and metmyoglobin (MetMb), a 1 cm layer was carefully removed from the breast muscle surface below the treated skin, cut into small pieces, frozen in liquid nitrogen, and stored at −80 °C until analysis (treatment groups: Tb-PAW, TRIS-buffer, untreated). Following the description of Bertram et al. [9], 3 g of the frozen meat and 7 mL of phosphate-buffered saline (PBS; Carl Roth) were homogenized (1 min at 30,000 rpm) with a homogenizer (MICCRA D-9, MICCRA GmbH, Heitersheim, Germany). The samples were then centrifuged (35,000× *g*; 30 min; 4 °C) and the supernatant was measured with a spectrophotometer (Evolution 201-UV–VIS-Spectrophotometer, Thermo Scientific) at 503, 525, 557, and 582 nm. DeoMb, OxyMb, and MetMb levels were calculated using the equations of Tang et al. [32].

### 2.12. Statistical Analysis of Data

Results from the three independent repeats were used for statistical analysis with SAS Enterprise Guide 7.1 (SAS Institute Inc., Cary, NC, USA). At first, the Shapiro-Wilks test for normality and Levene’s test for homogeneity of variances were applied. If the data were normally distributed and variance homogenous one-way analysis of variance (ANOVA) and Tukey’s range test (HSD) were applied. Otherwise, the Wilcoxon-two-sample test was used. The fixed factor of the analysis was the treatment group (Tb-PAW, TRIS-buffer, untreated). The data were visualized with GraphPad Prism (GraphPad Software, San Diego, CA, USA). *p*-values ≤ 0.05 were considered significant.

## 3. Results and Discussion

### 3.1. Bactericidal Efficacy of Tb-PAW on C. jejuni and E. coli

**Figure 2:**
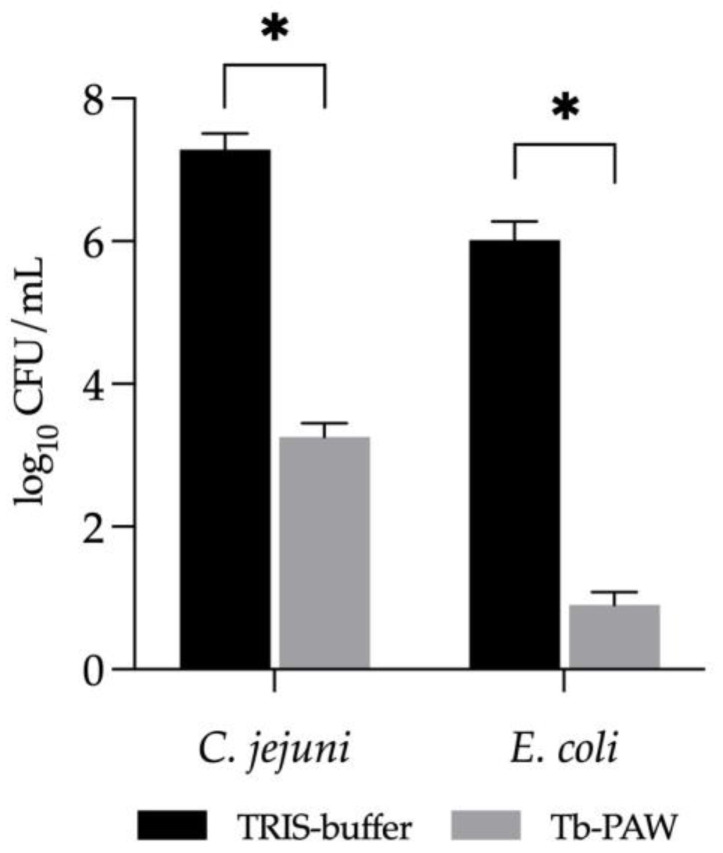
Inactivation of *Campylobacter (C.) jejuni* and *Escherichia (E.) coli* after 1 min of Tb-PAW treatment. Results represent the mean ± standard deviation. Significant differences are defined as * *p* ≤ 0.05. Tb-PAW = TRIS-buffered plasma-activated water.

At the outset of the experiments, the efficacy of Tb-PAW in inactivating *C. jejuni* and *E. coli* was assessed. The results are shown in Figure 2. Compared to the untreated TRIS-buffer control, Tb-PAW reduced the pathogens significantly (*p* ≤ 0.05) by approx. 4.20 ± 0.68 log_10_ CFU/mL (*C. jejuni*) and 5.12 ± 0.46 log_10_ CFU/mL (*E. coli*) after a 1 min exposure time.

Due to the application of different parameters such as power and activation time, working gases and flowrate, water source used, as well as different bacterial strains and levels of reactive species, PALs exhibit high variability in their antimicrobial activity [17,26,27,28]. This makes it more difficult to compare the present results with those of other studies. Both pathogens investigated were reduced by more than 4.0 log_10_ CFU/mL in a time that was short compared to other published trials [33,34]. For example, Zhao et al. [34] achieved a reduction of 3.0 log_10_ CFU/mL in *E. coli* after 30 min of treatment with PAW. Xiang et al. [33] treated *E. coli* O157:H7 for 6 min with PAW and only achieved a 3.70 log_10_ reduction. However, as far as we know, an inactivation of *C. jejuni* (in vitro) by PALs in general has not been published yet. Therefore, these are the first data that show a reduction of *C. jejuni* within a 1 min Tb-PAW treatment.

### 3.2. Microbial Analysis

**Figure 3:**
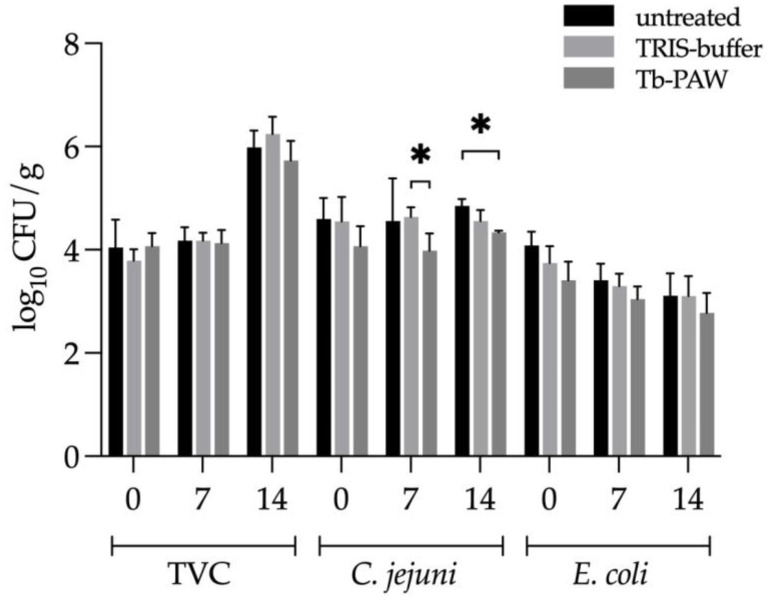
Reduction of the total viable count (TVC), *Campylobacter (C.) jejuni* and *Escherichia (E.) coli* on chicken carcasses either with or without Tb-PAW/TRIS-buffer treatment. Untreated and treated breasts (TVC) and thighs (*C. jejuni* and *E. coli*) with skin were stored in modified atmosphere packages (30% CO_2_, 70% N_2_) at 4 °C for 14 days. The samples from day 0 were analyzed on the packaging day, the others accordingly without re-treatment on days 7 and 14. Results represent the mean ± standard deviation. Significant differences are defined as * *p* ≤ 0.05. Tb-PAW = TRIS-buffered plasma-activated water.

**Figure 4:**
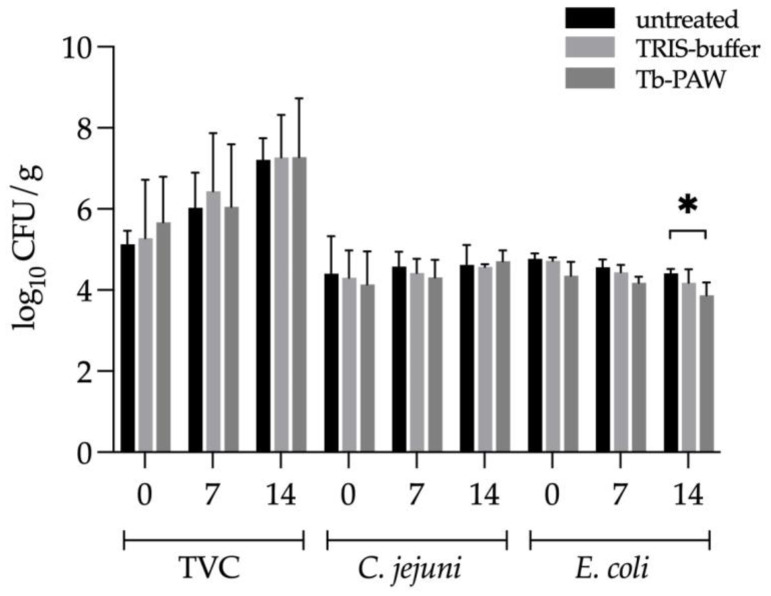
Reduction of the total viable count (TVC), *Campylobacter (C.) jejuni* and *Escherichia (E.) coli* on duck carcasses either with or without Tb-PAW/TRIS-buffer treatment. Untreated and treated breasts (TVC) and thighs (*C. jejuni* and *E. coli*) with skin were stored in modified atmosphere packages (30% CO_2_, 70% N_2_) at 4 °C for 14 days. The samples from day 0 were analyzed on the packaging day, the others accordingly without re-treatment on days 7 and 14. Results represent the mean ± standard deviation. Significant differences are defined as * *p* ≤ 0.05. Tb-PAW = TRIS-buffered plasma-activated water.

Spraying the Tb-PAW on the surface of chicken and duck thigh skin after inoculation with *E. coli* and *C. jejuni* and breast skin (without inoculation) followed by modified atmosphere packaging and storage over 14 days resulted in only a minor inhibitory effect on bacteria growth (Figure 3 and Figure 4). The Tb-PAW was able to achieve significant reductions of *C. jejuni* on the 7th and 14th day of storage after treatment of the chicken thigh skins. At day 7, a reduction was observed compared with the TRIS-buffer, whereas at day 14, the reduction only occurred between Tb-PAW and the untreated group. In contrast, *E. coli* and the TVC did not show improved reductions over the storage time after Tb-PAW treatment (Figure 3).

In contrast to chicken skin, the CFU from *E. coli* was significantly reduced by the Tb-PAW compared to the untreated samples on the 14th day of storage of the duck thigh skin. Considering the other storage days or the TVC and *C. jejuni* results, no significant effect of the Tb-PAW treatment could be obtained (Figure 4).

Furthermore, the number of CFU increased noticeably over the days of storage just as with the untreated samples. The chicken samples did not show an increase until 14 days, whereas the CFU increased steadily on the duck samples.

The antimicrobial effect of Tb-PAW on the skin of poultry carcasses yielded a lower reduction of *C. jejuni* and *E. coli* than in our in vitro study (Section 3.1). Currently, there are few studies that have investigated the reduction of microorganisms by PAWs specifically on poultry skin [25,35]. For example Kang et al. [25], who treated chicken breast muscles with PAW found significant antimicrobial effects but after a longer treatment period. The authors reduced *Pseudomonas deceptionensis* CM2 by approx. 1.0 log_10_ CFU/g by immersing chicken breasts in PAW for 12 min. However, this decrease was much lower compared the present study and compared to their previously published in vitro study [36] where they achieved a reduction of approx. 5.0 log_10_ CFU/mL after 10 min of PAW treatment. Sammanee et al. [35] described a significant reduction in microbial load of *E. coli* and *C. jejuni* after immersion of chicken samples in PAW (containing 60 ppm H_2_O_2_) for 15 min. However, the reduction by PAW was similar to the water treated control group for *E. coli*, but significant for *C. jejuni*, with reduction levels of 1.39 ± 1.10 log_10_ CFU/g.

According to the study by Xiang et al. [37], organic substances like proteins found in chicken breast skins may lead to a reduction in the antibacterial efficacy of PAW. This assumption is supported by the study of Royintarat et al. [17], who evaluated the antibacterial efficacy of PAW against *E. coli* on rough and smooth chicken skin. They found a decrease of only 0.56 log_10_ on rough skin (thickness of 4 mm) and only 0.35 log_10_ on smooth skin (thickness of 1 mm) after a 60 min PAW treatment at 40 °C. The organic matrix thus appears to be an important factor influencing the inactivation efficacy of PAL and CAP in general. As described by Fernandez et al. [38], this decreasing effect seems to become stronger when surface convolutions, as they occur on food surfaces or attachment sites, increase and microorganisms attach to their grooves or cavities.

The stability of PAW also plays a role in the inactivation efficiency against pathogens. In the study by Große-Peclum et al. [29], the stability of the Tb-PAW was already tested over a 24 h period. The Tb-PAW was stored at three different temperatures (7 °C, 21 °C, and 30 °C) and examined at four time points (4 h, 8 h, 12 h, and 24 h). This showed a dependence of PAW-stability on storage temperature and duration with an effective time stability of up to 12 h at all temperatures considered.

The spray treatment on the poultry skin and meat occurred on day 0 within this 12 h period. This ensured that time-dependent inactivation effects could be excluded.

In addition, combining Tb-PAW with other technologies such as ultrasound or heat could also increase inactivation efficiency against pathogenic and spoilage microorganisms [17,33].

### 3.3. Sensory Analysis

For chicken, the sensory properties tested were not significantly (*p* > 0.05) different between the treatment groups regardless of the storage day (Table 1). The test persons rated the overall properties on day 0 for both the untreated sample and Tb-PAW or TRIS-buffer as category 4 (good, slight deviations). On days 7 and day 14, the values after the Tb-PAW treatment remained in category 3 (satisfactory, slight deviations), whereas the values for the untreated sample and for the TRIS-buffer sample were classified in category 2 (less satisfactory, significant deviations). In duck, a significant reduction of the sensory results was observed at day 7 for the TRIS-buffer treated samples compared to the results of the other groups, which had similar sensory results.

Our results show that Tb-PAW does not negatively affect sensory parameters over a storage period of 14 days in chicken and duck samples compared to the other two treatment groups. The negative effect of the TRIS-buffer on day 7 of the duck experiments should not be overestimated, as it was only found on this storage day. Other authors who treated for example, chicken skin or muscle with PAW also found unchanged sensory characteristics [17] or deterioration [25]. According to Rahman et al. [13], the reactive oxygen and nitrogen species (RONS) contained in PAW can influence the biochemical and sensory properties of a food either positively or negatively. With the current settings of our Tb-PAW, there is no evidence of a negative impact on the sensory properties of the products after spraying.

### 3.4. Analysis of Meat Quality Parameters

Meat quality parameters were analyzed in both chicken and duck due to differences in their natural meat texture and skin structure. While the water, protein, and ash content of the muscles of both species are in comparable ranges (approx. 72–76%, 20–24%, and 1.0–1.3%, respectively), significant differences are found in the fat content. Intramuscularly about 1.3–1.6% fat content was determined in chicken, whereas as much as 2.5% fat was determined in duck. The collagen content was found to be somewhat lower in the duck (1.4%) than in the chicken (1.6–1.8%) [39,40,41,42,43]. However, not only the intramuscular composition, but also the different proportion of subcutaneous adipose tissue is likely to be relevant for the efficacy of PAW. In ducks, the proportion is significantly higher and can approach 30%, depending on sex and breed. In chickens, subcutaneous fat accounts for only 14% of the carcass [44,45]. Since the Tb-PAW is sprayed onto the skin, there could consequently be significant differences on the muscle under the skin (indirect effect) or on the exposed muscle (direct effect).

In the present study, the pH-value of chicken and duck breast meat was determined, as well as color measurements of the skin and muscle indirectly and directly after Tb-PAW/TRIS-buffer treatment. The color and pH-value are valuable criteria for determining the meat quality. In addition, color is an important indicator for consumers to visually judge the quality of fresh meat [13].

As shown in Table 1 and Table 2, there were no significant differences in pH-value and color results for the chicken carcasses on any of the storage days. Slightly, but significantly higher pH-values of the Tb-PAW treated samples were found in the duck carcasses compared to the untreated samples on day 7 and day 14. In addition, at day 0, significant differences of the lightness (L*) values were found between the Tb-PAW treated and the untreated duck breast skin samples. On day 0, the Tb-PAW treatment also resulted in lower a* values of the duck skin and meat (treated directly) compared to the other two treatment groups. On day 14, the yellowness/blueness (b*) values of the Tb-PAW indirectly treated meat were significantly higher compared to the untreated samples.

Considering the pH-values, the presented results are expected, as we tried to minimize any pH effect on the results in the present study by adjusting the Tb-PAW and the TRIS-solutions before treatment. Sammanee et al. [35] also evaluated the pH-value of chicken meat during a storage period of 10 days and found similar results between the PAW and untreated samples. In the study by Kang et al. [25], who treated chicken breasts without skin between 0 and 12 min with different PAWs, the initial pH-value for the control samples was 5.77. They described only a slight but significant decrease of the pH-value to approximately 5.70 after 12 min of treatment. This result might be attributed to the direct PAW treatment of chicken meat with an acidic pH-value. In contrast, in the present study, the Tb-PAW had a neutral pH-value and was sprayed on the skin, which could be a reason for unchanged or slightly increased pH-values.

As for the color, on one hand, many researchers have found no significant color change after PAW treatment of chicken breast [25], chicken meat and skin [17], or beef [46,47]. Qian et al. [48], on the other hand, found a lower a* value after PAW treatment, but they considered this change to be small and acceptable to consumers. Supporting this, the results in the present study also showed a decrease in a* values at day 0 (skin and muscle direct) in the duck carcasses. As described by Fröhling et al. [49]. The hydrogen peroxide produced in PAWs can react with myoglobin, giving treated meat a reduced red appearance. Furthermore, we found a slight increase in L* levels (skin, day 0) and b* levels (indirect muscle, day 14) in ducks but not in chicken. Sammanee et al. [35] also found increased L* levels on pork skin samples and increased b* levels (day 10) in pork red muscle, but no changes in chicken.

Overall, we can conclude that we merely found slight color differences after Tb-PAW treatment in our study, but these were perceived in the sensory evaluation.

### 3.5. Analysis of Myoglobin Redox Form Percentages and Antioxidant Activity

We analyzed the myoglobin redox forms and the antioxidant activity of chicken and duck breast muscles after indirect Tb-PAW and TRIS-buffer treatment (Table 3).

Antioxidants reduce reactive (oxygen, nitrogen) species thereby preventing or delaying cell damage (caused by the free radicals or unstable molecules). Thus, they help maintain the sensory properties of foods such as color, texture, freshness, odor and taste [50]. PAWs contain many radicals, for example, H_2_O_2_, which could decrease the amount of antioxidants and thus decrease the antioxidant activity of the samples. However, in the present study, the treatment with Tb-PAW had no significant reducing effect on the antioxidant activity of either chicken, or duck samples.

Myoglobin can occur in different states, namely as oxymyoglobin (OxyMb), deoxymyoglobin (DeoMb), or metmyoglobin (MetMb) and mainly influence the meat color. Here, the OxyMb provides the expected red appearance of the meat, due to the oxygenation of the myoglobin after oxygen contact. The DeoMb with its purple color is often found in vacuum stored meat. However, these two forms are so unstable that they can be converted to MetMb, which discolors the meat and gives it a brownish appearance [51]. In addition, duck meat has a much higher myoglobin content, therefore it appears much redder and chicken meat paler [52,53]. These color differences could result in different outcomes of the Tb-PAW application.

In this study, we analyzed the percentages of OxyMb (%OxyMb), MetMb (%MetMb), and DeoMb (%DeoMb) on the surface of the meat after treatment of the skin above these samples. No significant differences in %OxyMb, %DeoMb, and %MetMb were observed between all treatment groups on day 0 of storage in chicken as well as in duck. However, on day 14, in chicken Tb-PAW caused significantly lower %OxyMb and significantly higher %MetMb and %DeoMb results compared to the TRIS and untreated meat samples who had similar myoglobin redox form results. The effects of Tb-PAW on the myoglobin redox form percentages in duck were mainly similar on day 14, but the significant differences were between the untreated sample and Tb-PAW or TRIS-buffer (%OxyMb, %MetMb). For %DeoMb, only Tb-PAW and the untreated sample were significantly different.

To the best of our knowledge, no other studies have been published that investigated antioxidant activities of chicken or duck meat after PAW treatment. However, the present results are supported by studies that treated mung bean sprouts [24], grape extracts [54], and fresh-cut apples [55] with PAW and also found no effect on the antioxidant activities.

According to Mir et al. [56], the myoglobin content and muscle pH contribute to meat color and color defects. It seems that Tb-PAW (chicken and duck) and TRIS-buffer (only duck) cause a faster oxidation of %OxyMb to %MetMb under the skin than the untreated control. Compared to the lower %OxyMb and increased %MetMb and %DeoMb values, altered L*a*b* values of the indirectly treated meat sample would have been expected on day 14, but were not observed. Only in duck was a very slight deviation of b* values into the yellowish range, but this should not be overestimated. In the study of Astorga et al. [57], a PAW was tested on its storage quality of beef including the evaluation of myoglobin redox forms. They found no significant differences in %OxyMb for all PAW treatments compared to the untreated control.

The results show clear differences in the effect of Tb-PAW as well as TRIS-buffer in chicken and duck. This might be due to the species-specific characteristics of skin and muscle as described above.

## 4. Conclusions

The results of the present study show that Tb-PAW was able to reduce the amount of *C. jejuni* and *E. coli*. However, the in vitro experiment was associated with much higher reductions than the skin experiments. Nevertheless, promising reductions in bacterial counts of *C. jejuni* and *E. coli* were also observed on the surface of chicken and duck skin during the 14 day storage period after treatment. At the same time, Tb-PAW caused only minor significant changes in the physicochemical properties of chicken and duck skin and meat, which could not even be perceived in sensory analysis. However, the antimicrobial effect of Tb-PAW may vary greatly due to the influence of different parameters such as animal species or skin surfaces. The manufacture of Tb-PAW on a laboratory scale is currently associated with elevated operating and manufacturing costs, including the set-up of the plasma source (single-unit production), electricity for PAW production, and costs for the TRIS buffer. For example, the production of 1 L Tb-PAW under experimental conditions currently requires 0.5 kWh. However, in the case of an industrial application with optimized production processes, these costs could be significantly reduced (series production of the Tb-PAW) and a cost-effective and practical application of the Tb-PAW would be possible. In future studies, increasing the concentration of RONS in Tb-PAW, as well as combining Tb-PAW with other technologies such as ultrasound or heat, could enhance the reducing effect on the skin and thus reduce the risk of foodborne diseases such as campylobacteriosis.

## Figures and Tables

**Table 1:** Sensory acceptability (rated by three test persons) and the pH-values of chicken and duck breast meat and skin treated with or without Tb-PAW or TRIS-buffer depending on the day of storage (0, 7, 14) in modified atmosphere packages (30% CO_2_, 70% N_2_). Results represent the mean ± standard deviation. (N = 3).

Group	Storage Days	Treatment	Sensory Analysis	pH-Value
chicken	0	untreated ^1^	4.0 ± 0.7	6.0 ± 0.4
		TRIS ^2^	4.1 ± 0.6	5.9 ± 0.1
		Tb-PAW ^3^	4.0 ± 0.7	5.9 ± 0.1
	7	untreated	2.6 ± 0.3	5.7 ± 0.1
		TRIS	2.6 ± 0.4	5.7 ± 0.2
		Tb-PAW	3.2 ± 0.6	5.8 ± 0.1
		untreated	2.1 ± 0.2	5.7 ± 0.1
	14	TRIS	2.5 ± 0.3	5.8 ± 0.1
		Tb-PAW	3.1 ± 0.7	5.8 ± 0.1
duck	0	untreated	4.5 ± 0.2	5.6 ± 0.1
		TRIS	4.4 ± 0.3	5.6 ± 0.1
		Tb-PAW	3.9 ± 0.3	5.6 ± 0.1
	7	untreated	3.0 ± 0.2 ^a^	5.63 ± 0.1 ^b^
		TRIS	2.5 ± 0.2 ^b^	5.67 ± 0.1 ^a,b^
		Tb-PAW	3.4 ± 0.4 ^a^	5.67 ± 0.1 ^a^
	14	untreated	2.5 ± 0.6	5.65 ± 0.1 ^b^
		TRIS	2.4 ± 0.3	5.70 ± 0.1 ^a,b^
		Tb-PAW	3.0 ± 0.3	5.72 ± 0.1 ^a^

^a,b^ Different letters within the same column and the same storage day differ significantly (*p* ≤ 0.05). ^1^ Untreated breast fillets served as controls; ^2^ TRIS = TRIS-buffer; ^3^ Tb-PAW = TRIS-buffered plasma-activated water.

**Table 2:** Surface color of chicken and duck breast meat and skin treated with or without Tb-PAW or TRIS-buffer depending on the day of storage (0, 7, 14) in modified atmosphere packages (30% CO_2_, 70% N_2_). Results represent the mean ± standard deviation. (N = 3).

Group	Storage Days	Treatment	Skin Color			Meat Color Direct ^4^			Meat Color Indirect ^5^		
			L* ^6^	a* ^7^	b* ^8^	L*	a*	b*	L*	a*	b*
chicken	0	untreated ^1^	74.3 ± 0.3	4.1 ± 1.7	7.9 ± 1.2	60.1 ± 0.1	0.5 ± 0.5	4.0 ± 0.7	61.0 ± 0.9	1.6 ± 1.1	4.1 ± 0.9
		TRIS ^2^	74.2 ± 2.4	3.1 ± 1.4	7.6 ± 2.7	58.3 ± 2.2	−0.1 ± 0.4	2.8 ± 0.7	60.5 ± 1.0	0.5 ± 0.4	4.0 ± 0.5
		Tb-PAW ^3^	75.3 ± 0.9	1.7 ± 1.5	7.7 ± 1.6	59.4 ± 1.8	−0.1 ± 0.7	3.0 ± 1.8	60.9 ± 1.8	0.9 ± 0.9	4.0 ± 0.4
	7	untreated	73.6 ± 0.8	2.4 ± 0.9	6.0 ± 1.7	58.6 ± 1.6	1.0 ± 0.9	3.7 ± 1.5	59.1 ± 1.6	2.5 ± 1.3	4.0 ± 1.9
		TRIS	75.9 ± 1.2	2.8 ± 1.2	7.2 ± 1.2	58.8 ± 2.8	1.4 ± 0.1	2.8 ± 2.0	60.7 ± 2.1	2.7 ± 0.6	4.4 ± 1.8
		Tb-PAW	75.6 ± 1.8	2.6 ± 0.6	7.1 ± 1.5	58.2 ± 3.0	1.6 ± 0.3	3.1 ± 2.4	59.0 ± 1.7	4.1 ± 0.6	5.1 ± 1.3
		untreated	73.2 ± 2.4	2.1 ± 1.0	6.0 ± 0.9	58.4 ± 5.2	1.6 ± 1.6	3.2 ± 1.7	58.9 ± 3.6	3.8 ± 2.2	4.0 ± 1.0
	14	TRIS	74.9 ± 0.8	2.0 ± 1.1	7.0 ± 0.5	58.3 ± 2.5	0.6 ± 0.2	2.3 ± 1.1	59.5 ± 0.9	2.2 ± 0.4	4.4 ± 0.8
		Tb-PAW	74.8 ± 1.7	2.0 ± 1.0	6.9 ± 2.1	57.7 ± 2.2	1.2 ± 0.1	2.6 ± 1.1	58.2 ± 0.8	3.4 ± 0.6	4.6 ± 0.6
duck	0	untreated	76.6 ± 0.5 ^b^	4.5 ± 0.9 ^a^	16.4 ± 2.5	47.7 ± 1.4	11.7 ± 0.6 ^a^	6.4 ± 1.3	46.9 ± 1.5	11.8 ± 1.6	5.3 ± 2.6
		TRIS	76.7 ± 0.8 ^a,b^	3.0 ± 0.7 ^a^	14.6 ± 2.0	48.3 ± 1.3	10.7 ± 0.6 ^a^	6.5 ± 0.4	46.0 ± 1.2	11.3 ± 0.7	5.4 ± 0.6
		Tb-PAW	78.0 ± 0.4 ^a^	0.7 ± 0.3 ^b^	15.0 ± 1.4	47.3 ± 3.0	7.6 ± 0.8 ^b^	6.7 ± 0.3	47.7 ± 1.7	9.4 ± 1.5	5.2 ± 1.9
	7	untreated	77.1 ± 1.0	4.0 ± 1.0	14.1 ± 2.7	49.8 ± 2.8	13.0 ± 1.4	3.8 ± 1.9	48.8 ± 2.2	14.3 ± 1.3	3.1 ± 0.5
		TRIS	78.1 ± 1.2	3.3 ± 0.2	13.0 ± 1.7	48.3 ± 0.3	13.3 ± 1.5	4.3 ± 2.2	49.0 ± 1.8	14.0 ± 1.7	3.6 ± 1.5
		Tb-PAW	78.0 ± 0.4	3.4 ± 0.3	13.4 ± 2.2	48.0 ± 1.1	12.8 ± 0.8	4.6 ± 0.3	47.7 ± 1.9	15.5 ± 2.7	4.8 ± 1.1
	14	untreated	77.9 ± 4.1	5.7 ± 1.5	16.5 ± 2.8	48.5 ± 3.9	14.4 ± 0.9	4.1 ± 0.6	48.2 ± 5.2	15.8 ± 0.9	2.9 ± 0.9 ^b^
		TRIS	79.6 ± 5.8	5.5 ± 0.9	14.4 ± 2.6	52.0 ± 3.4	14.6 ± 0.8	5.0 ± 1.0	49.4 ± 2.4	15.5 ± 1.4	4.1 ± 1.9 ^a,b^
		Tb-PAW	79.1 ± 6.8	6.2 ± 1.2	14.7 ± 3.0	50.6 ± 3.2	14.3 ± 0.8	4.9 ± 0.8	48.4 ± 5.0	18.5 ± 2.2	5.8 ± 0.5 ^a^

^a,b^ Different letters within the same column and the same storage day differ significantly (*p* ≤ 0.05). ^1^ Untreated breast fillets served as controls; ^2^ TRIS = TRIS-buffer; ^3^ Tb-PAW = TRIS-buffered plasma-activated water. ^4^ direct = color values determined after application of Tb-PAW/TRIS-buffer directly to the meat surface; ^5^ indirect = color values determined after application of Tb-PAW/TRIS-buffer to the skin before analysis on the meat below the treated skin; ^6^ L* = lightness; ^7^ a* = red-green index; ^8^ b* = yellow-blue index.

**Table 3:** Percentages of oxymyoglobin (OxyMb), metmyoglobin (MetMb), and deoxymyoglobin (DeoMb) and the antioxidant activities of chicken and duck breast meat, treated on the skin above the meat with or without Tb-PAW or TRIS-buffer, depending on the day of storage (0, 14) in modified atmosphere packages (30% CO_2_, 70% N_2_). Results represent the mean ± standard deviation. (N = 3).

Group	Storage Days	Treatment	%OxyMb	%MetMb	%DeoMb	Antioxidant Activity ^4^
chicken	0	untreated ^1^	21.9 ± 2.6	55.6 ± 2.6	22.5 ± 0.4	24.2 ± 8.0
		TRIS ^2^	19.6 ± 1.9	56.9 ± 1.4	23.5 ± 1.4	23.1 ± 9.4
		Tb-PAW ^3^	19.1 ± 1.3	57.7 ± 0.8	23.2 ± 0.7	24.0 ± 11.6
		untreated	24.7 ± 4.8 ^a^	53.6 ± 3.2 ^b^	21.5 ± 1.9 ^b^	21.7 ± 9.3
	14	TRIS	21.5 ± 1.5 ^a^	56.5 ± 1.5 ^b^	21.9 ± 0.2 ^b^	23.3 ± 6.8
		Tb-PAW	14.6 ± 0.6 ^b^	60.8 ± 0.8 ^a^	24.7 ± 0.2 ^a^	24.0 ± 7.8
duck	0	untreated	68.9 ± 6.7	22.6 ± 5.3	6.4 ± 1.9	15.4 ± 1.8
		TRIS	63.5 ± 4.5	26.7 ± 2.8	7.9 ± 1.8	15.5 ± 1.4
		Tb-PAW	64.4 ± 3.3	25.7 ± 2.7	7.9 ± 1.0	16.0 ± 1.6
	14	untreated	68.8 ± 2.0 ^a^	20.7 ± 0.5 ^b^	8.2 ± 1.6 ^b^	14.0 ± 1.0
		TRIS	50.9 ± 9.6 ^b^	32.6 ± 5.2 ^a^	14.8 ± 4.7 ^a,b^	14.3 ± 2.1
		Tb-PAW	51.5 ± 8.9 ^b^	32.8 ± 6.1 ^a^	13.9 ± 3.0 ^a^	14.4 ± 2.4

^a,b^ Different letters within the same column and the same storage day differ significantly (*p* ≤ 0.05). ^1^ Untreated breast fillets served as controls; ^2^ TRIS = TRIS-buffer; ^3^ Tb-PAW = TRIS-buffered plasma-activated water. ^4^ in μmol Trolox eq. × g^−1^.

## Data Availability

Data is contained within the article or Supplementary Material.

## Supplementary Materials

The following supporting information can be downloaded at: https://www.mdpi.com/article/10.3390/foods12051113/s1, Figure S1: (a) Scheme of the plasma tube array to generate Tb-PAW (b) Scheme of the single plasma tube; Figure S2: U–I-characteristics of the plasma source at a power of approx. 400 W. Detailed information with regard to the plasma device and production of Tb-PAW from Große-Peclum et al. [29].
