# Supplementary material for: Using TRIS-Buffered Plasma-Activated Water to Reduce Pathogenic Microorganisms on Poultry Carcasses with Evaluation of Physicochemical and Sensory Parameters
Source: Foods. 2023 Mar 6;12(5):1113. doi: 10.3390/foods12051113 (PMC10000659; doi:10.3390/foods12051113)
Supplement: Supplementary file 1 [file foods-12-01113-s001.zip › foods-2234060-supplementary.pdf]

Supplementary Materials:

Using TRIS-Buffered Plasma-Activated Water to Reduce Pathogenic Microorganisms on Poultry Carcasses with Evaluation of Physicochemical and Sensory Parameters

Vanessa Große-Peclum, Lisa Siekmann, Carsten Krischek, Georg Avramidis, Christian Ochs, Wolfgang Viöl and Madeleine Plötz

For detailed information with regard to the plasma device and production of Tb-PAW we recommend the following section of the associated publication from Große-Peclum et al. [1].
Here you can find more illustrations and information about the technical production of the Tb-PAW as it was used in this study.

2. Materials and Methods

2.1. Plasma Device and Production of Tb-PAW

The Tb-PAW was produced by the faculty of engineering and health (HAWK, University of Applied Science and Arts, Göttingen, Germany) using an in-house development of the HAWK for the production of Tb-PAW based on the principle of a double-insulated DBD discharge. The complete setup for the generation of Tb-PAW consists of an arrangement of 10 individual "plasma tubes" which are integrated into an array (2 × 5) in a corresponding assembly (see Figure 1a).

The single plasma tube consists of an outer radially symmetrical silica tube (total length 300 mm, outer diameter 12 mm inner diameter 10 mm), wrapped with Cu-foil, which serves as the ground electrode (GND), and a centrally positioned ceramic tube (Al_2O_3) (Figure 1b). The ceramic tube has an outer diameter of 3 mm, an inner diameter of 1.6 mm and is filled with a brass rod (diameter 0.8 mm), the cavities are filled with brass powder. The ceramic tube acts as the high-voltage electrode. The chosen geometry results in a discharge gap of approx. 3.5 mm, which has a discharge length of approx. 100 mm. The discharge gap is streamed with pressure air as process gas at a gas volume flow rate of 5 $Lmin^{-1}$. This results in an average exposition time of the air to the discharge conditions of ≈0.1 s. The outer quartz tube protrudes approx. 5 cm into a beaker filled with deionized water. The distance between the end of the discharge section and the water surface is approx. 160 mm, so that the plasma exhaust contacts water after approx. 0.15 s after exiting the plasma zone.

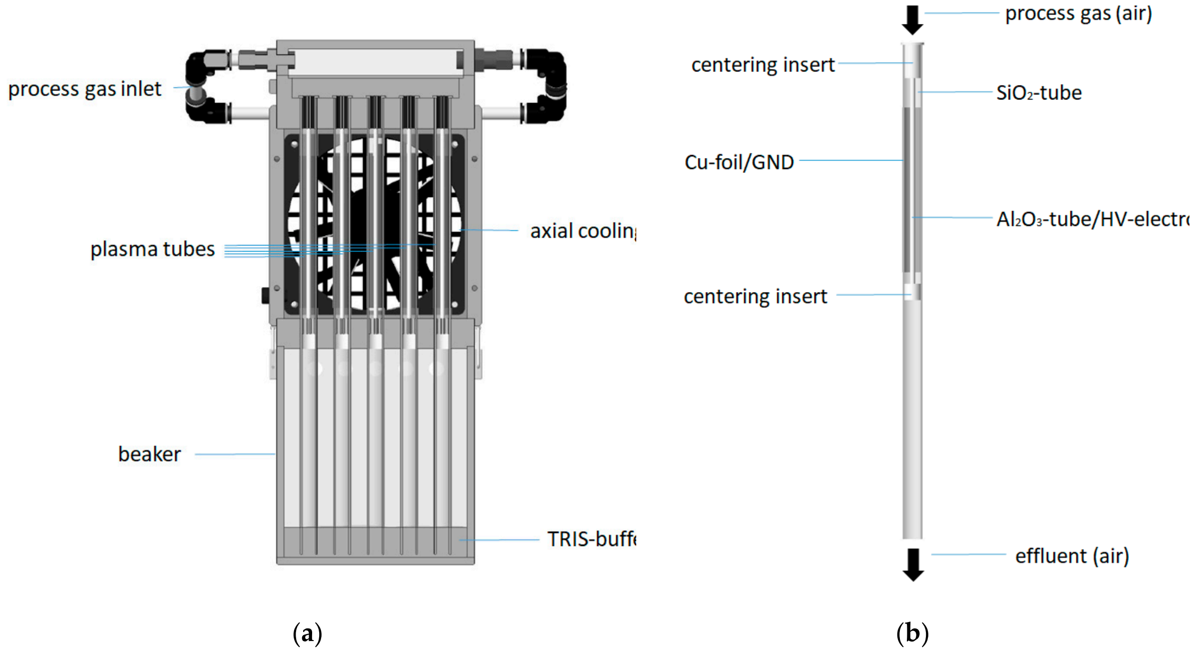

Figure 1. (**a**) Scheme of the plasma tube array to generate Tb-PAW **(b)** Scheme of the single plasma tube.

The high voltage power supply provides alternating pulses (U = 16.6 kV peak-peak) with a pulse repetition frequency of 17 kHz, a pulse duration of approx. 2 µs, an in-coupled power of approx. 400 W and a power density of approx. 6 W/cm^3 to the plasma-array. The characteristic U-I-envelope of the plasma source is depicted in Figure 2.

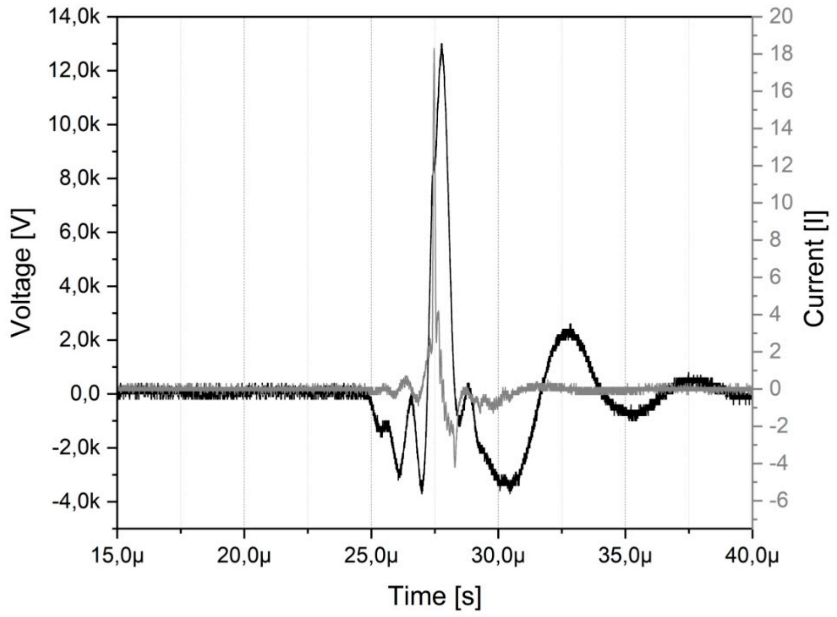

Figure 2. U–I-characteristics of the plasma source at a power of approx. 400 W.

The goal was to achieve a neutral pH-value of the Tb-PAW to allow an application on living animals in the future. Thus, TRIS-buffer (0.5 mol/L), made with TRIS(hydroxymethyl)aminomethan (TRIS,

Trometamol, ≥99.8%, VWR International, Darmstadt, Germany) and TRIS-HCl (TRIS(hydroxymethyl)aminomethan hydrochlorid, ≥99.0%, VWR), were treated with the plasma source for 20 min to obtain 250 mL Tb-PAW. The Tb-PAW and the (untreated, control) TRIS-buffer were transported at room temperature within 4 h after preparation to the Institute for Food Quality and Food Safety (LMQS, University of Veterinary Medicine Hannover, Germany). The experiments started within 4 to 5 h after preparation of the Tb-PAW. At first, the ph-values of the TRIS-buffer control (7.6 ± 0.2) and the Tb-PAW sample (7.3 ± 0.2) were measured with a pH-meter (Jenway, Cole-Parmer, Stone, Staffordshire, ST15 OSA, UK) equipped with a glass electrode (InLab Semi-Micro, Mettler Toledo, Gießen, Germany). For the experiments, the pH-values of all TRIS-buffer samples were adjusted to the respective pH-value of the Tb-PAW in order to exclude a pH-effect. Additionally, prior to the transport to the LMQS a Reflectoquant (Merck KGaA, Darmstadt, Germany) was used to measure the concentrations of nitrate anions (NO_3^-; approx. 5540 mg/L) and nitrite anions (NO_2^-; approx. 440 mg/L), as well as hydrogen peroxide (H_2O_2; approx. 4.5 mg/L) concentrations in the Tb-PAW.

References

1. Große-Peclum, V.; Siekmann, L.; Krischek, C.; Avramidis, G.; ten Bosch, L.; Harms, M.; Ochs, C.; Ortmann, R.; Hoedemaker, M.; Ahlfeld, B.; et al. An In Vitro Model Using TRIS-Buffered Plasma-Activated Water to Reduce Pathogenic Microorganisms Involved in Digital Dermatitis Infection in Cattle. *Applied Sciences* **2022**, *12*, 12325, doi:10.3390/app122312325.
